# A Data Science-Based Analysis Points at Distinct Patterns of Lipid Mediator Plasma Concentrations in Patients With Dementia

**DOI:** 10.3389/fpsyt.2019.00041

**Published:** 2019-02-11

**Authors:** Robert Gurke, Semra Etyemez, David Prvulovic, Dominique Thomas, Stefanie C. Fleck, Andreas Reif, Gerd Geisslinger, Jörn Lötsch

**Affiliations:** ^1^Institute of Clinical Pharmacology, University Hospital of Frankfurt, Goethe-University, Frankfurt, Germany; ^2^Department of Psychiatry, Psychosomatic Medicine and Psychotherapy, University Hospital of Frankfurt, Goethe-University, Frankfurt, Germany; ^3^Fraunhofer Institute for Molecular Biology and Applied Ecology IME, Branch for Translational Medicine and Pharmacology TMP, Frankfurt, Germany

**Keywords:** dementia, data science, lipids, biomarker, machine learning

## Abstract

Based on accumulating evidence of a role of lipid signaling in many physiological and pathophysiological processes including psychiatric diseases, the present data driven analysis was designed to gather information needed to develop a prospective biomarker, using a targeted lipidomics approach covering different lipid mediators. Using unsupervised methods of data structure detection, implemented as hierarchal clustering, emergent self-organizing maps of neuronal networks, and principal component analysis, a cluster structure was found in the input data space comprising plasma concentrations of *d* = 35 different lipid-markers of various classes acquired in *n* = 94 subjects with the clinical diagnoses depression, bipolar disorder, ADHD, dementia, or in healthy controls. The structure separated patients with dementia from the other clinical groups, indicating that dementia is associated with a distinct lipid mediator plasma concentrations pattern possibly providing a basis for a future biomarker. This hypothesis was subsequently assessed using supervised machine-learning methods, implemented as random forests or principal component analysis followed by computed ABC analysis used for feature selection, and as random forests, k-nearest neighbors, support vector machines, multilayer perceptron, and naïve Bayesian classifiers to estimate whether the selected lipid mediators provide sufficient information that the diagnosis of dementia can be established at a higher accuracy than by guessing. This succeeded using a set of *d* = 7 markers comprising GluCerC16:0, Cer24:0, Cer20:0, Cer16:0, Cer24:1, C16 sphinganine, and LacCerC16:0, at an accuracy of 77%. By contrast, using random lipid markers reduced the diagnostic accuracy to values of 65% or less, whereas training the algorithms with randomly permuted data was followed by complete failure to diagnose dementia, emphasizing that the selected lipid mediators were display a particular pattern in this disease possibly qualifying as biomarkers.

## Introduction

Accumulating evidence supports a high relevance of lipid molecules, including so-called lipid mediators (1), for the regulation of many different biological processes (2–4). Lipidomics has therefore become one of the latest omics-technologies used in the search for biomarkers (1), i.e., defined characteristics of biological systems measured as indicators of normal biological processes, pathogenic processes, or responses to an exposure or (therapeutic) intervention (5). A possible advantage of lipidomics over some other omics-technologies, such as genomics, is the close temporal linkage of lipid marker concentrations with individual clinical phenotypes (4, 6). Lipidomics includes several thousands of different molecules (7) found in biological fluids at highly variable concentrations, assayed using untargeted approaches (2, 6, 7) which aim to quantity the whole lipidome in a single analytical run but lack sensitivity for molecules in the low concentration range and selectivity for differences among isomeric or isobaric molecules (3, 7). On the other hand targeted approaches are used. They are focused on a limited number of analytes at comparatively high sensitivity and selectivity (3).

Consistent to the nearly ubiquitous involvement of lipid mediators in physiological and pathophysiological processes, lipid markers have been linked to neurological disorders, cancer, the metabolic syndrome, pain (1, 2, 6–8), and other clinical settings. Neuropsychiatric disorders have also been proposed as novel applications for lipidomics based diagnostics (9). This agrees with altered lipid profiles observed in dementia or Alzheimer's disease (10, 11), depression (12), and bipolar disorder (12). The present investigation was designed to gather information needed to develop a prospective biomarker, using a targeted lipidomics approach covering different lipid mediators. Therefore, a data-driven approach was preferred to an explicit hypothesis about suitable psychiatric settings and lipid mediators.

## Methods

### Subjects and Study Design

The study followed the Declaration of Helsinki and was approved by the Ethics Committee of the Medical Faculty of the Goethe—University Frankfurt am Main, Germany (protocol number 425/14). Informed written consent was obtained from all subjects. Patients with depression (*n* = 20), bipolar disorder (*n* = 20), attention-deficit hyperactivity disorder (ADHD; *n* = 12), or dementia (*n* = 16) were consecutively recruited from outpatients and inpatients of the Department of Psychiatry of the University Hospital Frankfurt am Main, Germany. Controls (*n* = 26) were enrolled from medical students and staff members of the hospital who routinely reported to the institutional occupational health service. Inclusion criteria were age ≥ 18 years, for patients, a clinically verified diagnosis according to ICD-10 criteria and for controls, no current medical condition queried by medical interview, and no drug intake for at least 1 week except contraceptives, vitamins, and L-thyroxin.

Demographic data including time since diagnosis and current disease-specific medication are summarized in Table 1. Of the patients with bipolar disorder (F31, including manic, hypomanic, mixed, or depressive episode), *n* = 19 received mood stabilizing medication, including lithium, valproate, lamotrigine, carbamazepine, and antipsychotics. Patients fulfilling diagnostic criteria for moderate or severe depressive episode (F32, F33) received antidepressant treatment with selective serotonin reuptake inhibitors (SSRIs) (*n* = 8), dual serotonergic drugs (trazodone) (*n* = 1), serotonin, and noradrenalin reuptake inhibitors (*n* = 6), mirtazapine (*n* = 2), tricyclic antidepressants (*n* = 1), or monoamineoxidase-inhibitors (*n* = 1). In addition, *n* = 6 patients received mood stabilizers. Of the patients with attention deficit disorder (F90.0 and F98.8), *n* = 2 were treated with stimulants approved for this disease (methylphenidate, atomoxetine) at the time of blood sampling. The dementia group (*n* = 16) was comprised of patients with probable Alzheimer's dementia (*n* = 10; G30.1 and F00.1) and mild cognitive impairment (MCI) (*n* = 6; F06.7). Of these patients, *n* = 2 received antidementia drugs at time of blood sampling (memantine or cholinesterase-inhibitors). In most dementia and ADHD cases blood sampling was done shortly after diagnosis, which explains why only few patients were already on disease-specific medication. Moreover, no antidementia drugs are approved for the treatment of MCI, which additionally explains why a large fraction of this group was untreated.

**Table 1:** Demographic parameters of the subjects, temporal disease characteristics, and medication.

**Diagnosis**	**Parameter**	**Mean**	***SD***	**Count (*n*)**
Depression	Sex [count] m/f	9/11		
	Age [y]	43.1	14	20
	BMI [kg/m^2^]	26.2	6.9	18
	Duration^*^ (months)	8.7	11.2	
	Medicated patients (*n*) ^**^	19		
Bipolar disorder	Sex [count] m/f	11/9		
	Age [y]	43.7	12.7	20
	BMI [kg/m^2^]	26.4	5.4	19
	Duration^*^ (months)	1.9	2.7	
	Medicated patients (*n*) ^**^	19		
ADHD	Sex [count] m/f	6/6		
	Age [y]	38.6	15.6	12
	BMI [kg/m^2^]	29.1	9.2	11
	Duration^*^ (months)	0.9	0.5	
	Medicated patients (*n*) ^**^	2		
Dementia	Sex [count] m/f	10/6		
	Age [y]	69.9	9.3	16
	BMI [kg/m^2^]	26.2	3.7	16
	Duration^*^ (months)	5.1	15.4	
	Medicated patients (*n*) ^**^	2		
Healthy	Sex [count] m/f	15/11		
	Age [y]	39.6	13.1	26
	BMI [kg/m^2^]	23.6	2.2	26

* Duration: for depression and bipolar disorder: average duration of current episode; for ADHD and Dementia: average time since diagnosis.

** *Medicated patients: number of patients treated with disease-specific medication at time of blood sampling (Depression: treatment with antidepressants, Bipolar disorder: treatment with mood stabilizers; ADHD: treatment with methylphenidate or atomoxetine; Dementia: treatment with cholinesterase-inhibitors or memantine)*.

### Lipid Mediator Plasma Concentration Analysis

A venous blood sample (2.7 ml) was collected into a K3EDTA blood collection tube (Sarstedt, Nürmbrecht, Germany) and centrifuged at 2,000 g for 15 min at 4°C within 30 min after sample collection. Plasma was separated and frozen at −80°C until assay. A total of *n* = 41 different lipid mediators and other metabolites was analyzed from the plasma samples. Plasma concentration analyses were performed using liquid chromatography-electrospray ionization-tandem mass spectrometry (LC-ESI-MS/MS) as described in detail in the Supplementary Material. The selected methods included sphingolipids (sphingosine, sphingosine-1-phosphate, sphinganine-1-phosphate, C16 sphinganine, C18 sphinganine, C24 sphinganine, C24:1 sphinganine, Cer14:0, Cer16:0, Cer18:0, Cer20:0, Cer24:0, Cer24:1, GluCerC16:0, GluCerC18:0, GluCerC18:1, GluCerC24:1, LacCerC16:0, LacCerC18:0, LacCerC24:0, LacCerC24:1), lysophosphatidic acids (LPA16:0, LPA18:0, LPA18:1, LPA18:2, LPA20:4), endocannabinoids [arachidonoyl ethanolamide (AEA), oleoyl ethanolamide (OEA), palmitoyl ethanolamide (PEA), 1-arachidonoyl glycerol (1-AG), 1-arachidonoyl glycerol (2-AG), docosahexaenoyl ethanolamide (DHEA)], nucleosides (cytidine, deoxycytidine, guanosine, thymidine, uridine), nucleotides [guanosine triphosphate (GTP), adenosine triphosphate (ATP)] and cyclic nucleotides [3′,5′-cyclic guanosine monophosphate (3′,5′-cGMP), 3′,5′-cyclic adenosine monophosphate (3′,5′-cAMP)]. Because lipid mediators represent the major part of investigated metabolites hereafter the lipid mediators will be used as general term comprising all investigated metabolites.

### Data Analysis

Data analysis was performed using the R software package [version 3.4.4 for Linux; http://CRAN.R-project.org/; (13)] on an Intel Core i9® computer running on Ubuntu Linux 18.04.1 64-bit). The acquired parameters, subsequently called “features,” initially included *d* = 41 lipid mediators assayed from the participants' venous blood plasma. The analysis was performed in three main steps comprising (i) data preprocessing, (ii) hypothesis generation using unsupervised lipid mediator pattern analysis to assess whether the lipid mediator patterns contained a cluster structure separating patients with particular diagnoses from other patients, and (iii) hypothesis testing using supervised lipid mediator pattern analysis aimed at identification of lipid mediators important for diagnosis group assignment.

#### Data Preprocessing

The original data set comprised d = 41 lipid mediators. In six mediators (sphingosine, Cer14:0, guanosine, 1-AG, 2-AG, DHEA), more than 20% of the measurements across all diagnoses were below the lower limit of quantification (LLOQ) while most of the other measurements of these mediators were only narrowly above LLOQ. This was considered as an indication of insufficient assay sensitivity for the present set of plasma samples and therefore, these lipid mediators were excluded from further analysis. Following published recommendations (14), values below the validated LLOQ were verified by the responsible analyst and values above the limit of detection were used in the data set. Using this technique, seven values below LLOQ for Cer16:0 and 12 values below LLOQ for 3′,5′ -cGMP were included giving full data sets for both analytes. For deoxycytidine only eight out of 17 values below LLOQ were replaceable leaving the remaining nine as missing values.

Data preprocessing included (i) data transformation, (ii) outlier detection and elimination, (iii) imputation of missing data, and (iv) corrections for possible confounding influences of the patients' age, sex, or BMI. Data transformation was performed following exploration of the data distribution of the *d* = 35 features (i.e., the remaining lipid mediators) by means of Kolmogorov-Smirnov tests (15), applied to the original data and after application of log, square root, or reciprocal data transformation. Kolmogorov-Smirnov tests obtained *p* < 0.05 in 12, 2, 5, and 3 parameters when not transformed or when log, square root or reciprocal transformation was applied, respectively. As log-transformation is most frequently advised for transformations of blood-concentration data (16), this was chosen for d = 33 parameters. For the two remaining parameters, ATP and GTP, in which log transformed data significantly differed from normal distribution (*p* = 0.044 and *p* = 0.00044, respectively), reciprocal transformation was applied, which is a common transformation for plasma concentrations applied, for example, to creatinine (17). This resulted in non-significant results of Kolmogorov-Smirnov tests also for ATP and GTP (*p* = 0.737 and *p* = 0.327, respectively).

Subsequent to data transformation, outlier in both directions were detected by means of Grubbs tests (18). Specifically, each outlier was replaced with a missing value. The procedure was iteratively repeated as long as significant results of Grubbs tests were obtained. A total of 9 outliers was detected and replaced with missing values in the 35 lipid mediators. These calculations were performed using the R library “outliers” [https://cran.r-project.org/package=outliers; (19)]. Following outlier elimination, the data set included 18 missing values. Imputation was performed using the k-nearest neighbor algorithm (20). These calculations were performed using the R library “DMwR” [https://cran.r-project.org/package=DMwR; (21)]. Subsequently, a 94 × 35 sized complete matrix of lipid mediator concentrations was available for further analysis.

Differences with respect to age, body mass index (BMI), or sex among the diagnostic groups (four psychiatric diagnosis and controls) were assessed by means of univariate analysis of variance. This resulted in significant age differences (df = 4.85, *F* = 16.49, *p* = 3.84 × 10^−10^) did not differ significantly (df = 4,85, *F* = 2.206, *p* = 0.0751). The sex distribution was equal among diagnostic groups as indicated by a non-significant χ^2^ test (22) (χ^2^ = 1.3443, df = 4, *p* = 0.85). As age between groups potential confounders of subsequent results, the correlation of lipid mediator plasma concentrations with age was explored by calculating Pearson correlation coefficients (23). In the controls (age range 21–67 years), 17 lipid parameters were found to be significantly correlated with age. Subsequently, robust linear regressions of lipid parameter concentrations vs. age were calculated in the controls only. Slopes and intercepts were used to correct, in the whole data set, the concentrations of lipid mediators found to be age-correlated in the controls, for age. During this procedure, the respective parameters were also normalized to the median age of subjects. This eliminated the age correlation as indicated by non-significant Pearson correlation tests in the control subgroup.

#### Hypothesis Generation Using Unsupervised Lipid Mediator Pattern Analysis

Hypothesis generation with respect to which of the candidate psychiatric diseases was associated with altered lipid mediator patterns was pursued via structure detection in the preprocessed data. Specifically, structures hinting at subgroups of subjects were sought in the data space *D* = {(*x*_*i*_)|*x*_*i*_ ∈ *X, i* = 1, …, *n*} comprising plasma concentrations of *d* = 35 lipid mediators acquired in *n* = 94 subjects. Data structures were assessed by means of unsupervised methods including (i) Ward hierarchical clustering, (ii) emergent self-organizing maps of artificial neurons (ESOM), and (iii) classical principal component analysis.

Following the standardization of all features into a numerical range of [0,…,100], the data space was explored for possible subgroups of subjects sharing similar lipid mediator concentration patterns by means of hierarchical clustering using the Ward algorithm (24) and the Euclidean distance. To identify the optimum number of clusters in the data space, cluster stability scores were computed addressing the consensus in cluster assignments across multiple runs of the clustering algorithm on data sets created by repeated random resampling from the original data set (25). That is, the stability scores capture the average proportion of observations not placed in the same cluster during the repeated runs (26). Clustering, including stability assessments was performed using the progeny algorithm (27), which selects the optimum cluster number that renders the most stable clustering by evaluation of clustering stability starting with an initial clustering of the full dataset, followed by bootstrapping (28) and repetitive clustering. The idea is that a meaningful valid cluster shouldn't disappear easily if the data set is changed in a non-essential way (29) such as by drawing a random subsample of the cases. Cases belonging together to the same cluster should keep shared cluster membership when the case composition of the data set is slightly modified. During resampling, the algorithm randomly sampled feature values with replacement to construct new samples, so-called “progenies,” rather than directly sampling existing samples as with common algorithms. A number of k = [2,…,5] clusters was tried using 10 progenies in 10 randomly created datasets and 100 iterations, which corresponds to the defaults of the R package “progenyClust” [https://cran.r-project.org/package=progenyClust; (30)] used for these calculations. To obtain standard deviations of the stability measures, the procedures were repeated 10 times. The final number of clusters was chosen on the basis of both criteria offered in the progeny clustering algorithm, i.e., the stability score criterion and in addition, the so-called “greatest gap” criterion that selects the cluster number that renders the greatest difference in stability score compared to its neighboring numbers (27).

Lipid mediator-based cluster structures were again explored by means of unsupervised machine learning. Specifically, a topology-preserving self-organizing map (SOM) of the Kohonen type (31, 32) was trained with the lipid mediator concentration information. SOM are based on a topology-preserving projection of high-dimensional data points x_i_ ϵ R^D^ onto a two-dimensional self-organizing network consisting of a grid of neurons. The neural network consisted of a two-dimensional toroid grid of so-called neurons with 50 rows and 80 columns [*n* = 4,000 units, for SOM size determination, see (33)]. Each neuron holds, in addition to a position vector on the two-dimensional grid, a further vector carrying “weights” of the same dimensions as the input dimensions. The weights were initially drawn randomly from the sets of data variables and subsequently, adapted to the data during the learning phase with 30 epochs. Following training of the neural network, a SOM was obtained that represented the subjects on a two-dimensional toroid map as the localizations of their respective “best matching units” (BMU) that are neurons on the grid that which after SOM learning carried the vector that was most similar to a subject's data vector. To obtain clusters, an extension of the Kohonen SOM aimed at enhancing a cluster structure and consisting of the so-called U-matrix (34), was added. The U-matrix displays the distances between the neurons in the high dimensional space as a third dimension onto the two-dimensional SOM projection grid. Large heights indicated a large gap in the data space, whereas low U-heights indicated that the points were close to each other in the data space, indicating structure in the data set. Using a topographic map analogy of coloring, on the U-matrix appeared valleys, ridges and basins that enhanced the visibility of clusters. For example, a “mountain range” separated two regions indicating clusters which appeared as “valleys” surrounded by the “mountains.” These calculations were performed using the R package “Umatrix” [https://cran.r-project.org/package=Umatrix; (35)].

To explore associations between clusters and diagnosis groups, the respective contingency tables were analyzed with respect to overrepresentation or underrepresentation of diagnosis groups in clusters. Specifically, the permutation distributions of the two-way tables were obtained and the sums of squares of the Pearson residuals were calculated as the test statistic of independence (36). Test significance was assessed using Pearson's χ^2^ test (22). This allowed to associate particular diagnoses with Ward or U-matrix based clusters. Clusters in which a particular clinical diagnosis was overrepresented provided hypotheses for further assessments. These calculations were done using the R library “vcd” [https://cran.r-project.org/package=vcd; (37)].

As an independent approach for internal validation of the suitability of the data transformations and of the detected structure in the data, principal component analysis (PCA) (38) was performed. PCA (38) uses a rotation of the data, to project the data to a subspace of so-called principal components. The first principal component has the largest possible variance in the data. Each succeeding orthogonal component is chosen for the highest possible remaining variance. PC-corr analysis (39) provides an algorithm that permits to find the best results of a PCA. PC-corr uses various data transformations and analyses group separations by quantitative evaluations expressed as *p*-value, AUC and AUPR, using any types of normalization and dimension. As it calculates various quality measures for every combination of PC, normalization and centering, it allows the optimal selection of PC for data projection and was therefore a suitable method to verify both, the applied data transformation and results indicating that the data support a group structure. This analysis was performed using an R script provided with the description of the PC-corr analysis [pccorrv2.R, https://github.com/biomedical-cybernetics/PC-corr_net; (39)].

#### Hypothesis Testing Aimed at Identification of Most Relevant Lipid Mediators

Following identification of a hypothesis about which of psychiatric diseases was associated with altered lipid marker concentrations, subsequent analysis aimed at identification of the most relevant lipid mediators among the *d* = 35 candidate features. Hence, dimension reduction or of feature selection (40) procedures were implemented as usually conducted when a large number of candidate variables are present in order to narrow the focus to the most relevant lipid mediators. Of note, this analytical step aimed at selecting a subset of the existing features without a transformation further to the simple numerical transformation based on the distribution of each individual lipid marker. By contrast, feature extraction was not the final target, i.e., transforming the existing features (lipid mediator plasma concentrations taken at a single time point) into some derived variable with lower dimensions. This is not contradicted by the intermediate use of PCA, which can be used for feature extraction; however, in the present implementation, the PCs were used to obtain the most informative lipid mediators but they were not further used in subsequent analyses (see below). Feature selection was performed using (i) machine learning techniques implemented as random forests and (ii) by assessing the PCA results for features that provided most relevant contributions for data variance explanation.

Random forests (41, 42) is a method of supervised machine learning (43) aimed at finding a mapping from inputs *x* to output *y*, given a labeled set of input-output pairs *D* = , composed of class assignments ∈ *Y* = *N* comprising diagnoses or groups of diagnoses, and of values ∈ *X* ⊂ comprising the *d* features, respectively plasma concentrations of lipid mediators, acquired from *n* > 0 cases (subjects). Random forests is a method of ensemble learning and creates sets of different, uncorrelated, and often very simple decision trees with conditions of features shown as vertices and classes as leaves. The splits of the features are random and the classifier relates to the majority vote for class membership provided by a large number of decision trees. Random forest learning was performed following the concept of a nested cross-validation analysis (44). Specifically, using Monte-Carlo (45) resampling, the original data set was split into a training (2/3 of the data) and test (1/3 of the data) data set, using the R library “sampling” [https://cran.r-project.org/package=sampling; (46)]. Subsequently, random forest analysis was performed using randomly drawn sub-samples from the actual training sample and finally, the forest was applied to the actual test sample. The calculations were done using the R library “randomForest” [https://cran.r-project.org/package=randomForest; (47)]. Precedent hyperparameter testing had indicated that *sqrt(d)* parameters for each tree, which is the standard procedure implemented in the R library, provided a similar classification performance as alternative settings. In addition, using this setting the out-of-bag error remained at its lowest level of 27.77% starting from forests sizes of 210 trees; however, forest sizes of 1,500 trees were used in further analyses. As a measure against overfitting, this random forest analysis was repeated on 1,000 different training and test data sets, randomly drawn from the original training data set.

Identification of the most relevant lipid mediators was based on the mean decrease in classification accuracy, when the respective feature was excluded from random forest building. The extent of this decrease indicated the importance of the particular feature in a single random forest run. Hence, after each random forest analysis, the values for the mean decrease in tree classification accuracy, when the feature was excluded from random forest analysis, were subsequently submitted to computed ABC analysis (48). This is a categorization technique for the selection of the most important subset among a larger set of items. It was chosen since it fitted the basic requirements of feature selection using filtering techniques (40). Thus, it easily scales to very high-dimensional datasets, is computationally simple and fast, and independent of the classification algorithm. ABC analysis aims to divide a set of data into three disjointed subsets called “A,” “B,” and “C” (49). Subset “A” contains the most profitable features (50, 51) and was therefore, chosen for classifier establishment. These calculations were done using our R package “ABCanalysis” [http://cran.r-project.org/package=ABCanalysis; (48)]. For each of the 1,000 runs using Monte-Carlo (45) resampling to split the original data set into a training (2/3 of the data) and test (1/3 of the data) data set, the size and members of the ABC set “A” were retained. The final size of the feature set was equal to the most frequent size of the set “A” in the 1,000 runs, and the final members of the feature set were chosen in decreasing order of their appearance in the ABC set “A” among the 1,000 runs.

Applying the most suitable PCA parameter settings as identified by means of PC-corr analysis, PCA was used for feature selection. Specifically, features were analyzed with respect to how much of the variance of a given PC was explained by the original variable. This was obtained by calculating Pearson's correlation (52) and taking the square *r*^2^ of the correlation coefficient between the variable and the PC. Subsequently, values of *r*^2^ were submitted to ABC analysis as described above. Again, For each of 1,000 runs using Monte-Carlo (45) resampling to split the original data set into a training (2/3 of the data) and test (1/3 of the data) data set, the size and members of the ABC set “A” were retained. The final size of the feature set was equal to the most frequent size of the set “A” in the 1,000 runs, and the final members of the feature set were chosen in decreasing order of their appearance in the ABC set “A” among the 1,000 runs.

Following the identification of lipid markers relevant to the group structure among lipid mediator plasma concentrations that had emerged from data structure detection analysis during hypothesis generation, the sets obtained by either random forests or PCA, followed by computed ABC analysis, were compared. A final set was created from the set intersection as this was considered to comprise lipid markers that by two independent methods had emerged as most relevant in the present context.

The selected lipid mediators were assessed with respect to their utility for class assignment, i.e., to correctly predict different groups of patients as established in the hypothesis generation step of the analysis. Therefore, a mapping of the input space, given by the selected features prepared during the precedent analytical steps, to the output space, given by the classes obtained during hypothesis generation, was performed. The mapping was addressed by random forests classifier building applied on the original data set and on a negative control data set obtained by random permutation of the lipid mediator concentration pattern in the respective training data subsets. The expectation was to observe a prediction of the subjects' class membership that was better when using the original lipid mediator concentration pattern than when using the permuted patterns, which should provide a classification performance not superior to guessing. In addition, the procedure was repeated with randomly chosen sets of lipid mediators that were not selected as best suited for classification in the preceding analytical steps, with the expectation that this selection provided a poorer classification performance than when using the selected features. Furthermore, the procedure was run again with the full set of *d* = 35 lipid mediators to further establish that the best suited features had been selected for the reduced set.

As random forests had been used for feature selection, which could have provided sets of lipid mediators only suited for class assignment when this particular method was used, analyses of the classification performance were repeated using further methods of supervised machine-learning comprising k-nearest neighbors (kNN) (20), support vector machines (SVM) (53), multilayer perceptrons (54), and naïve Bayesian classifiers. This provided different types of classifiers. Specifically, while random forests confers a decision tree based ensemble learning classifier, kNN provide a prototype based classifier, support vector machines and multilayer perceptrons use supervised-artificial neuronal-network based classifiers, however, differently constructed, and naïve Bayesian classifiers represent probabilistic classifiers based on the Bays theorem (55) and including independency assumptions.

During kNN classification the entire labeled training dataset is stored while a test case is placed in the feature space in the vicinity of the test cases at the smallest high dimensional distance. The test case receives the class label according to the majority vote of the class labels of the *k* training cases in its vicinity. In the present implementation, the size of *k* was established in resampling experiments, and considering the small group size in the cohort, with *k* set at 3. These calculations were performed using the R package “KernelKnn” [Mouselimis L, https://cran.r-project.org/package=KernelKnn]. Support vector machines are supervised learning methods that classify data mainly based on geometrical and statistical approaches employed for finding an optimal decision surface (hyper-plane) that can separate the data points of one class from those belonging to another class in the high-dimensional feature space (53). These analyses were done using the R library “kernlab” [https://cran.r-project.org/package=kernlab; (56)].

Furthermore, a perceptron (54) was built from artificial neurons that are provided with several input channels, a processing level, and an output level that connects a neuron to one or multiple other artificial neurons. In the present analyses, a multilayer perceptron was used with a number of input neurons equaling the number of lipid mediators selected during the feature selection step, three hidden layers composed of 35 neurons, and the output layer comprising as many neurons as classes the output space of the analyses. The number of neurons in the hidden layers was determined during exhaustive assessments of perceptrons with one to three hidden layers sized between two neurons each and the maximum number of lipid mediators included. Standard back propagation was used for the learning function. The analyses were done using the R library RNNS [https://cran.r-project.org/package=RSNNS; (57)]. Finally, naïve Bayesian classifiers were used that provide the probability that a data point being assigned to a specific class calculated by application of the Bayes' theorem (55). The calculations were done using the R package “klaR” [https://cran.r-project.org/package=klaR; (58)]. The performances of all classifiers were assessed using the test data set drawn up at the start of the data analysis and comprised the calculation of standard measures of test performance (e.g., sensitivity, specificity, precision, positive and negative predictive values, F1 measure, balanced accuracy) as implemented in the R library “caret” [https://cran.r-project.org/package=caret]. In addition, for the area under the ROC curve (AUC-ROC) and the area under the precision-recall curve (AUPRC) were calculated for the results obtained with the selected lipid mediators and the original data, using the R libraries “pROC” [Robin X, https://cran.r-project.org/package=pROC; (59)].

## Results

**Figure 1:**
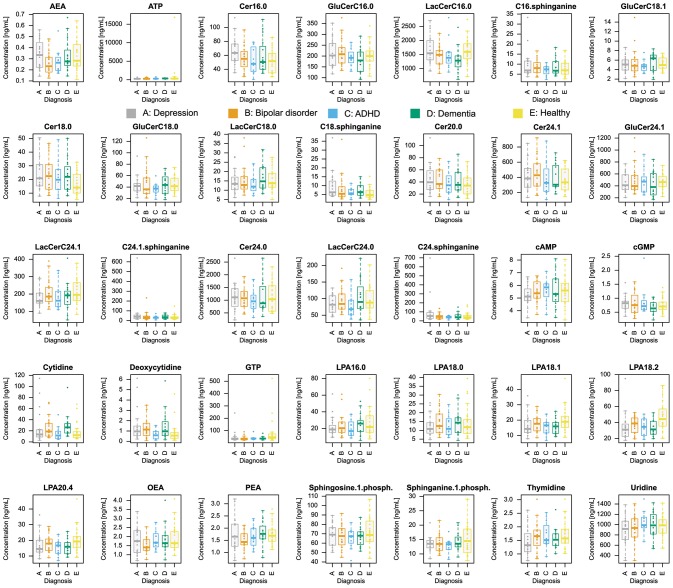
Plasma concentrations of *d* = 35 lipid mediators (raw data). The data are shown in alphabetical order of lipid mediator names and for each mediator, separately for group membership to the five diagnoses (coded as A to E for depression, bipolar disorder, ADHD, dementia, or controls, respectively). The widths of the boxes are proportional to the respective numbers of subjects per group. The quartiles and medians (solid horizontal line within the box) are used to construct a “box and whisker” plot. Single data are shown as dots. The whiskers add 1.5 times the interquartile range (IQR) to the 75th percentile or subtract 1.5 times the IQR from the 25th percentile and are expected to include 99.3% of the data if normally distributed. The figure has been created using the R software package [version 3.4.4 for Linux; http://CRAN.R-project.org/; (13)].

Plasma concentrations of *d* = 35 lipid mediators (Figure 1) were available from *n* = 94 subjects with the clinical diagnoses depression (*n* = 20), bipolar disorder (*n* = 20), ADHD (*n* = 12), dementia (*n* = 16), or healthy (*n* = 26).

### Hypothesis Generation Using Unsupervised Analysis of Lipid Mediator Patterns

**Figure 2:**
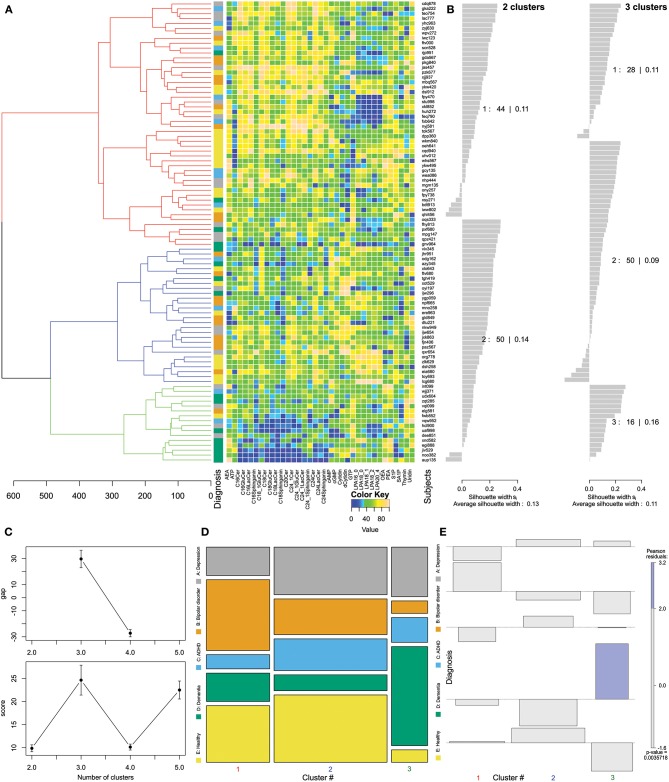
Data structure found obtained by means of Ward clustering (24) of the data space of *d* = 35 plasma lipid mediator concentrations acquired in *n* = 94 subjects. **(A)** Cluster dendrogram showing three clusters, and matrix plot of the transformed and age corrected data rescaled to a range of [0,…,100] shown in the adjacent matrix plot displaying the transformed concentrations of *d* = 35 lipid mediators (columns) acquired from *n* = 94 subjects (rows) with color coding for the scaled data. **(B)** Silhouette plots (60) for 2 or 3 clusters, which indicate how near each sample is to its own relative to the neighboring clusters. Positive values indicate that the sample is away from the neighboring clusters while negative values indicate that those samples might have been assigned to the wrong cluster because they are closer to neighboring than to their own cluster. The low silhouette coefficients of 0.11 or 0.13, however, indicate a weak cluster structure. **(C)** The optimal cluster number was identified based on cluster stability criteria assessed using the progeny algorithm (27, 30). The optimal cluster number was chosen based on the two criteria: “greatest score” and “greatest gap” **(C)** top and bottom, respectively, which both indicated three clusters. The error bars indicate standard deviations calculated from 10 runs of the progeny algorithm. **(D)** Mosaic of the contingency table between diagnosis groups (ordinate) and clusters (abscissa). The size of the cells is proportional to the number of subjects included. **(E)** Association plot visualizing the residuals of an independence model for the diagnosis vs. clusters contingency table (61). Each cell of the contingency table is represented by a rectangle that has (signed) height proportional to the signed contribution to Pearson's χ for the cell and width proportional to the square root of expected counts corresponding to the cell. Hence, the area of each box is proportional to the difference in observed and expected frequencies. The rectangles in each row are positioned relative to a baseline indicating independence, i.e., if the observed frequency of a cell is greater than the expected one, the box rises above the baseline, and falls below otherwise. Each diagnosis (lines) is plotted vs. the Ward derived clusters (columns) as a result of a contingency table analysis, indicating the relative representations of each cluster in across the tree nodes. The Pearson residuals are colored according to a perceptually uniform Hue-Chroma-Luminance (HCL) given at the right margin of the association plot (62). The figure has been created using the R software package [version 3.4.4 for Linux; http://CRAN.R-project.org/; (13)]. Specifically, for drawing the silhouette plots, the R library “cluster” was used [https://cran.r-project.org/package=cluster; (63)] and, tree and association plots were drawn using the R package “vcd” [https://cran.r-project.org/package=vcd; (37)] including the “strucplot” framework (37) and residual-based shadings (36), and the results of progeny cluster number detection are the graphical output of the R library “progenyClust” [https://cran.r-project.org/package=progenyClust; (30)].

Applying the progeny algorithm (27), three clusters were identified as the most stable solution of hierarchical clustering of the lipid mediator plasma concentration pattern observed in the 94 subjects (Figure 2). This result was based on both, the “greatest score” criterion (i.e., the most stable clustering solution as detailed out in the methods section) and the “greatest gap” criterion (i.e., the criterion that selects the cluster number that renders the greatest difference in stability score compared to its neighboring numbers) (27). The diagnostic groups were unequally distributed among clusters (Pearson χ^2^ = 22.843, df = 8, *p* = 0.00357). Nevertheless, the distribution of diagnoses often agreed with the expectation from a random distribution (Figure 2). The only significant deviation from this expectation was observed with the diagnosis of dementia that was significantly overrepresented in cluster #3. Thus, results of Ward clustering suggested that patients with the diagnosis of dementia display a lipid plasma mediator pattern that differs from that of all other included patients and controls.

**Figure 3:**
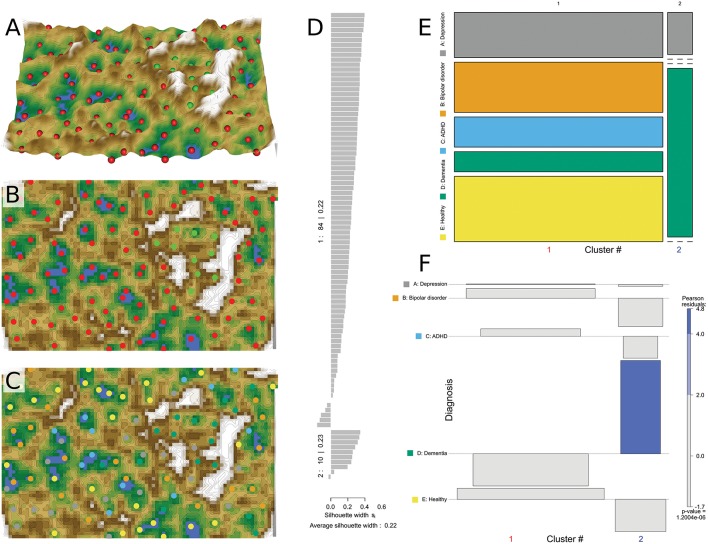
Data structure found obtained by means of SOM based clustering of the data space of *d* = 35 plasma lipid mediator concentrations acquired in *n* = 94 subjects. **(A)** 3-dimensional display of the U-matrix visualization of distance based structures of the lipid mediator plasma concentration pattern (transformed and age corrected data rescaled to a range of [0,…,100]) observed in *n* = 94 subjects. The figure has been obtained using a projection of the data points onto a toroid grid of 4,000 neurons where opposite edges are connected. The dots indicate the so-called “best matching units” (BMUs) of the self-organizing map (SOM), which are those neurons whose weight vector is most like the input. A single neuron can be the BMU for more than one data point or subject, hence, the number of BMUs may not be equal to the number of acquired measurements. The U-Matrix was colored as a geographical map with brown (up to snow-covered) heights and green valleys with blue lakes. Valleys indicate clusters and watersheds indicate borderlines between different clusters. The BMUs are colored according to the two clusters identified on the basis. **(B)** Top view of the same U matrix, **(C)** Top view of the same matrix with BMUs color coded for the diagnosis groups. This indicates that the small cluster separated from the other data by a circular “mountain ridge” contained in particular patients with dementia. **(D)** Silhouette plot (60) for the 2 cluster solution. Positive values indicate that the sample is away from the neighboring clusters while negative values indicate that those samples might have been assigned to the wrong cluster because they are closer to neighboring than to their own cluster. The silhouette coefficient of 0.22 indicates better cluster sedation than observed with the Ward based clusters (Figure 2). **(E)** Mosaic of the contingency table between diagnosis groups (ordinate) and clusters (abscissa). The size of the cells as proportional to the number of subjects included. **(F)** Association plot visualizing the residuals of an independence model for the diagnosis vs. clusters contingency table (61). Each cell of the contingency table is represented by a rectangle that has (signed) height proportional to the signed contribution to Pearson's χ^2^ for the cell and width proportional to the square root of expected counts corresponding to the cell. Hence, the area of each box is proportional to the difference in observed and expected frequencies. The rectangles in each row are positioned relative to a baseline indicating independence, i.e., if the observed frequency of a cell is greater than the expected one, the box rises above the baseline, and falls below otherwise. Each diagnosis (lines) is plotted vs. the Ward derived clusters (columns) as a result of a contingency table analysis, indicating the relative representations of each cluster in across the tree nodes. The Pearson residuals are colored according to a perceptually uniform Hue-Chroma-Luminance (HCL) given at the right margin of the association plot (62). The figure has been created using the R software package [version 3.4.4 for Linux; http://CRAN.R-project.org/; (13)]. Specifically, the U matrix was drawn using our R package “Umatrix” [https://cran.r-project.org/package=Umatrix; (35)], for drawing the silhouette plots, the R library “cluster” was used [https://cran.r-project.org/package=cluster; (63)] and tree and association plots were drawn using the R package “vcd” [https://cran.r-project.org/package=vcd; (37)] including the “strucplot” framework (37) and residual-based shadings (36).

A trained self-organizing map of the Kohonen type was obtained with the U-matrix providing a visible separation of a small cluster from most of the neurons (Figure 3). Specifically, using the topographical map analogy a circular mountain range was visible that surrounded a “valley,” which indicates the emergence of two clusters in the data (64). Superimposing onto the cluster structure the class labeling into diagnostic groups indicated the separate small cluster was mainly populated by patients with dementia, whereas the data structure did not further coincide with the structure of the diagnoses groups. Indeed, although the diagnostic groups were unequally distributed among the two clusters (Pearson χ^2^ = 32.99, df = 8, *p* = 1.2 × 10^−6^), the distribution of diagnoses agreed with the expectations from a random distribution except for the diagnosis of dementia that was significantly overrepresented in cluster #2 (Figure 3). Thus, results of SOM-based clustering suggested that patients with the diagnosis of dementia display a lipid plasma mediator pattern that differs from that of all other included patients or controls.

**Figure 4:**
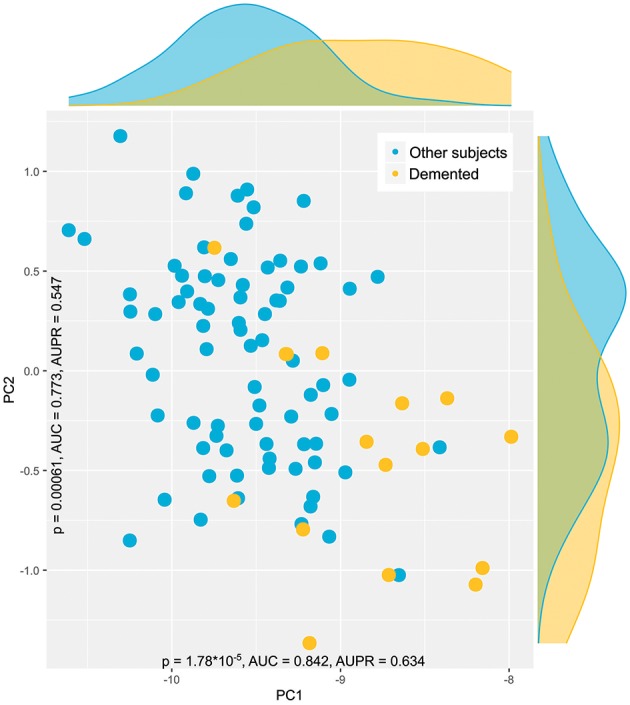
Data structure found in the input space of *d* = 35 lipid mediator plasma concentration acquired from patients with dementia (yellow dots) or from patients with either bipolar disorder, depression, ADHD, or enrolled as healthy controls (blue dots). The data structure has been obtained by means of principal component analysis on the log-transformed data as suggested by the results of the PC-corr analysis (39). The PCA plot associated to this analysis shows the sample separation in the first and second component (PC1 vs. PC2) as explaining most of the variance in the data. In addition, the group-wise marginal distributions of the data are shown. The figure has been created using the R software package [version 3.4.4 for Linux; http://CRAN.R-project.org/; (13)] and the library “ggplot2” [https://cran.r-project.org/package=ggplot2; (65)].

Results of unsupervised data analysis aimed at hypothesis generation, thus consistently indicated that of the five diagnostic groups, lipid mediator plasma concentration patterns in patients with dementia differed from those found in any other enrolled group of subjects. Applying the PC-corr algorithm to the back-transformed data, i.e., to the antilog or reciprocals of the transformed, outlier curated and age corrected data, suggested a non-centered PCA and log transformation of the data (Supplementary Table 1). This produced a significant segregation of the patients with dementia from other subjects along the first dimension (PC1). Indeed, the sample segregation along PC1 had *p*-value < 0.001, AUC-ROC of 0.84, AUC-PR of 0.77, and explained 99% of the variance (Figure 4). Hence, log transformation of most of the data, as it had resulted from data exploration during the preprocessing step, was supported by the results of the PC-corr analysis.

**Figure 5:**
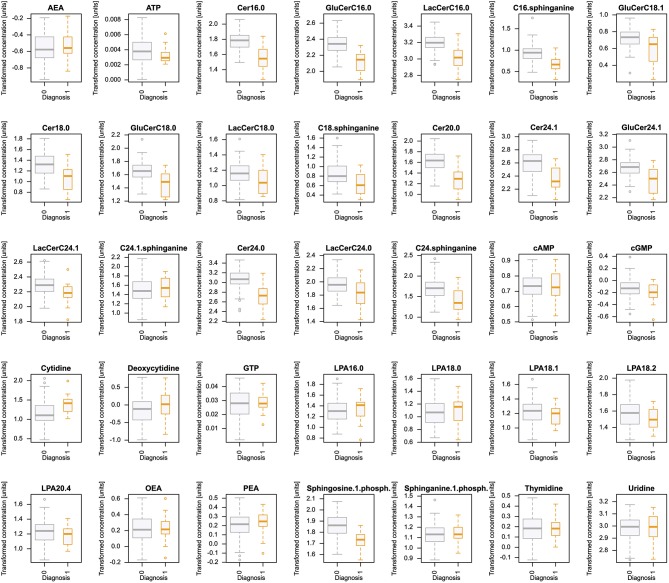
Plasma concentrations of *d* = 35 lipid mediators (transformed and age corrected data) acquired from patients with dementia (yellow) or from patients with either bipolar disorder, depression ADHD or enrolled as healthy controls (gray). The data are shown in alphabetical order of lipid mediator names and for each mediator, separately for group membership to either or not the diagnosis of dementia (dementia = 1, others = 0). The widths of the boxes are proportional to the respective numbers of subjects per group. The quartiles and medians (solid horizontal line within the box) are used to construct a “box and whisker” plot. Single data are shown as dots. The whiskers add 1.5 times the interquartile range (IQR) to the 75th percentile or subtract 1.5 times the IQR from the 25th percentile and are expected to include 99.3% of the data if normally distributed. The figure has been created using the R software package [version 3.4.4 for Linux; http://CRAN.R-project.org/; (13)].

Based on these results the hypothesis was generated that lipid mediator concentration patterns differ between patients with dementia and all other subjects enrolled in this study (Figure 5). Subsequent data analyses were designed accordingly.

### Identification of Most Relevant Lipid Mediators

**Figure 6:**
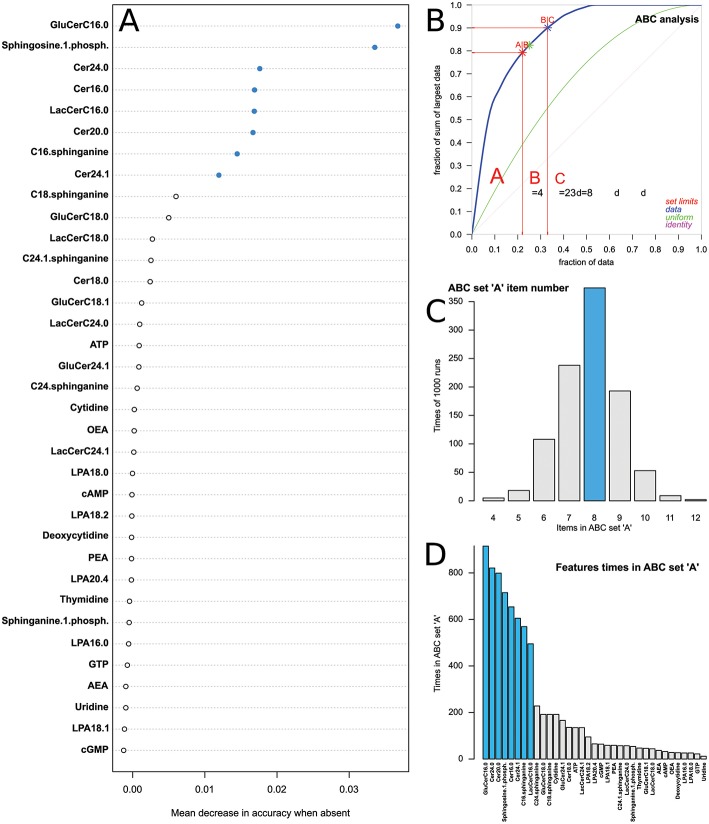
Feature selection based on random forests analysis followed by computed ABC analysis, for the diagnosis of dementia that was consistently identified to display a lipid mediator plasma concentration pattern that differed from that of the other subjects. **(A)** Display of the mean decrease in classification accuracy when the respective feature (lipid mediator concentration) is excluded from the random forest analysis. The plot displays one typical example out of the analyses of 1,000 bootstrap resampled data subsets. **(B)** Subsequent to random forest-based feature ranking, the mean decrease in accuracy associated with each item was submitted to computed ABC analysis, which is an item selection procedure aiming at identification of most profitable items from a larger list of items. The ABC plot (blue line) shows the cumulative distribution function of the mean decreases in accuracy, along with the identity distribution, *x*_*i*_ = constant (magenta line), i.e., each feature contributes similarly to the classification accuracy [for further details about computed ABC analysis, see (48)]. The red lines indicate the borders between ABC sets “A,” “B,” and “C.” Only set “A” containing the most profitable items was selected as the diagnosis-relevant subsets. **(C)** Bar plot of the number of lipid mediators found in ABC set A during the 1,000 runs. **(D)** Bar plot of the features' importance in descending order of their appearance in ABC set “A” during the 1,000 runs. As set “A” had most frequently a size *d* = 8, the eighth features (blue bars) found most frequently in set “A” were selected as the most relevant lipid mediators for the diagnosis of dementia in this model. The figure has been created using the R software package [version 3.4.4 for Linux; http://CRAN.R-project.org/; (13)] and our R package “ABCanalysis” [http://cran.r-project.org/package=ABCanalysis; (48)].

For the 1,000 runs of random forest building followed by ABC analysis of the feature importance measure, i.e., of the mean decrease in classification accuracy when the lipid mediator was omitted from forest building, the ABC set “A” had sizes of 4–12 items. The most frequent size |{ABC set A}| = 8 lipid mediators, i.e., the most profitable items from the set of candidate features (Figure 6), was chosen for further analysis. The eight lipid mediators that during the 1,000 runs have been most frequently members of ABC set “A” comprised GluCerC16:0, Cer24:0, Cer20:0, sphingosine-1-phosphate, Cer16:0, Cer24:1, C16 sphinganine, and LacCerC16:0. Applying the parameters identified by the PC-corr analysis to the PCA, a significant segregation of sample emerged along the first dimension (PC1) that explained 99% of the variance. Hence, the main criterion of feature importance was chosen to be the squared Pearson correlation coefficient of the lipid maker concentration data with the loadings on the first principal component. For the 1,000 runs of PCA followed by ABC analysis of this feature importance measure, the ABC set “A” had sizes of 9–15 items. The most frequent size |{ABC set A}| = 13 lipid mediators, i.e., the most profitable items from the set of candidate features (Figure 6), was chosen for further analysis. The 13 lipid mediators that during the 1,000 runs have been most frequently members of ABC set “A” comprised Cer16:0, GluCerC18:0, Cer20:0, Cer24:1, GluCerC24:1, Cer24:0, Cer18:0, GluCerC16:0, C16 sphinganine, LacCerC18:0, C24 sphinganine, GluCerC18:1, and LacCerC16:0. From these results, a final set of lipid mediators relevant for the generated hypothesis was created from the set intersection of the random forests and PCA derived features assigned by ABC analyses to set “A” that contains the most relevant items of a set. This intersection comprised *d* = 7 markers, i.e., GluCerC16:0, Cer24:0, Cer20:0, Cer16:0, Cer24:1, C16 sphinganine, and LacCerC16:0.

Validation of the suitability of the selected lipid mediators was performed using five different methods comprising (i) random forests, (ii) k nearest neighbors (kNN), (iii) support vector machines, (iv) multilayer perceptrons, and (v) naïve Bayesian classifiers. They were applied on (i) the set of most relevant lipid mediators applied to the original data, (ii) equally sized sets of lipid mediators randomly chosen from those not included in the set of most relevant mediators, and (iii) the full set of *d* = 35 lipid mediators. All sets of mediators were trained (i) with the original data and (ii) with data in which the lipid mediator concentrations had been randomly permuted along the subjects, i.e., disrupting the association between lipid mediator and class label.

**Figure 7:**
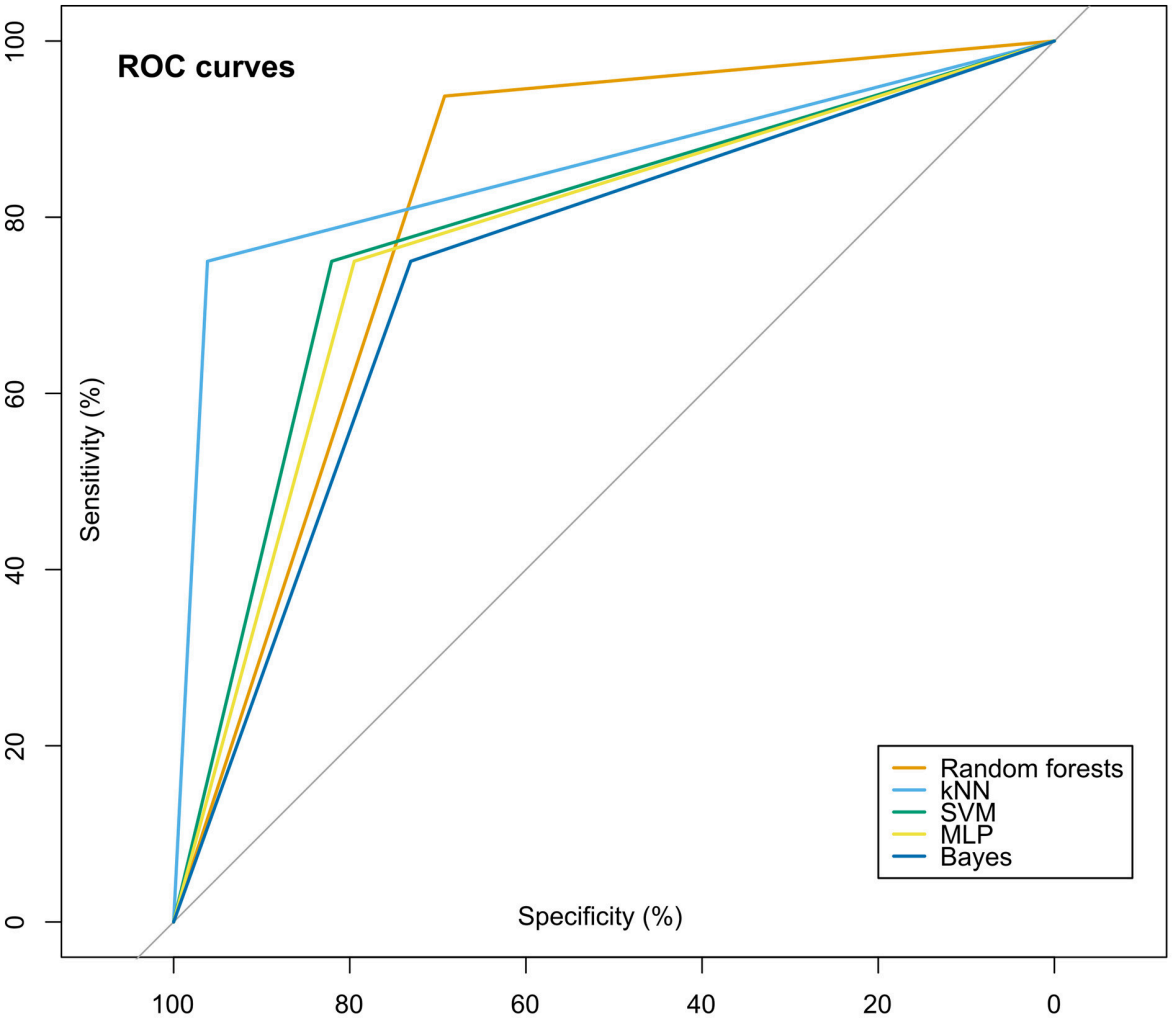
Receiver operating characteristic (ROC) curves for the classifiers. For this example, the full data set was used for the prediction of trained algorithms implemented as random forests, k-nearest neighbors (kNN) support vector machines (SVM) multilayer perceptron [MLP or naïve Bayesian classifiers (Bayes)]. The figure has been created using the R software package [version 3.4.4 for Linux; http://CRAN.R-project.org/; (13)] and the R package “pROC” [https://cran.r-project.org/package=pROC; (59)].

Across these 3 × 5 × 2 validation scenario (three data sets, five algorithms, original/permuted data), it was consistently found that when using the selected feature set of lipid mediators relevant to the present classification problem into subjects with dementia vs. other subjects enrolled in this study, the performance in correct class assignment was similar or sometimes better to that obtained with the full feature set. The performance measures are shown in Table 2; the mean areas under the ROC curves were 0.78, 0.77, 0.8, 0.8, and 0.8 obtained with random forests, k-nearest neighbors, support vector machines, multilayer perceptrons, and naïve Bayesian classifiers, respectively, during the 1,000 iterations with randomly resampled disjoint training and test data subsets. An example is shown in Figure 7. The performance measures were substantially better than those obtained when used similarly sized sets of lipid mediators chosen among those that had not passed the feature selection step. By contrast, when training the algorithms with randomly permuted data, the class association was not better than by flipping a coin, i.e., was obtained at a mean balanced accuracy of around 50%.

**Table 2:** Performance measures of classifiers obtained using different machine-learned methods [random forests (RF), k-nearest neighbors (kNN) support vector machines (SVM)] on the data set comprising *d* = 35 lipid mediators.

	**Selected features**										**Non-selected**										**All features**									
	**Original data**					**Permuted data**					**Original data**					**Permuted data**					**Original data**					**Permuted data**				
**Performance measure [%]**	**RF**	**kNN**	**SVM**	**MLP**	**NB**	**RF**	**kNN**	**SVM**	**MLP**	**NB**	**RF**	**kNN**	**SVM**	**MLP**	**NB**	**RF**	**kNN**	**SVM**	**MLP**	**NB**	**RF**	**kNN**	**SVM**	**MLP**	**NB**	**RF**	**kNN**	**SVM**	**MLP**	**NB**
Sensitivity	79	78	86	86	84	51	51	50	51	51	66	62	67	67	66	51	49	50	52	50	81	68	83	79	80	50	51	50	51	51
Specificity	77	75	73	74	75	49	52	50	48	49	66	63	64	63	64	51	48	51	50	51	76	78	73	73	74	49	50	49	49	50
Pos Pred Value	80	79	79	79	79	50	51	48	49	49	69	65	67	67	67	51	48	50	51	50	80	79	78	77	77	50	50	49	50	50
Neg Pred Value	82	81	87	87	85	50	53	52	51	52	67	64	68	68	68	52	49	51	52	51	83	74	84	81	82	50	52	49	51	51
Precision	80	79	79	79	79	50	51	48	49	49	69	65	67	67	67	51	48	50	51	50	80	79	78	77	77	50	50	49	50	50
Recall	79	78	86	86	84	51	51	50	51	51	66	62	67	67	66	51	49	50	52	50	81	68	83	79	80	50	51	50	51	51
F1	77	76	81	81	80	57	57	57	57	56	66	62	66	66	66	53	53	54	54	52	78	71	79	76	77	53	54	54	54	53
Prevalence	50	50	50	50	50	50	50	50	50	50	50	50	50	50	50	50	50	50	50	50	50	50	50	50	50	50	50	50	50	50
Detection Rate	39	39	43	43	42	26	25	25	26	26	33	31	33	34	33	25	25	25	26	25	40	34	41	40	40	25	25	25	26	25
Detection Prevalence	51	51	56	56	54	51	49	50	52	51	50	49	51	52	51	50	51	49	51	49	52	45	55	53	53	50	51	50	51	51
Balanced Accuracy	78	77	80	80	80	50	52	50	50	50	66	62	65	65	65	51	49	50	51	50	78	73	78	76	77	50	50	50	50	50

*The classification task to identify, from the lipid mediator plasma concentration pattern, patients with dementia among a mixed cohort of patients with depression, bipolar disorder, ADHD, or healthy controls. Results represent the means of the test performance measures from 1,000 model runs using random splits of the data set into training data (2/3 of the data set) and test data (1/3 of the data set). The classifiers were trained on the original data set and again on a randomly permuted training data, which served as negative control to detect possible overfitting*.

## Discussion

A role of lipids and lipid mediators, i.e., bioactive lipid molecules involved in different biological signaling events (1), has already been shown for several diseases; however, a possible exploitation as biomarkers in the detection of diseases or to monitor the efficacy of medical treatment can be challenging. As lipidomics includes several thousands of molecules found in biological materials at very different concentration ranges (7), published concentration data are limited and comparisons among different studies may be difficult due to missing quality assurance measures, differences in the lower limit of quantification, inadequate method validation, or non-standardized sample collection procedures. Often, changes in concentrations of single lipid mediators are small and therefore, it can be assumed that no single molecule but a complex pattern of concentration changes of different molecules will be identified as biomarker (66). This poses challenges on the data analysis triggering the preference for bioinformatics methods such as machine learning that have already been applied successfully to the association of diseases with lipidomic pattern changes (2, 66–68).

In the present analyses, machine-learning was mainly used for knowledge discovery and hypothesis generation. The data analytical process mimicked the creation of a biomarker having this in mind as a subsequent project based on present results. The focus was on hypothesis generation through data exploration in a typical data science project that includes the import and tidying of the data, data transformations, visualizations and modeling, and ends with the communication of the results (69). The algorithms at the end of the analysis served for the verification of the generated hypothesis. Given small study sample, a classifier for immediate use as a biomarker was not expected to arise. Its creation will require independent data in an adequately powered study, which is now possible since a clear hypothesis has been generated during the present analysis.

Indeed, data exploration was used with its goal of generating promising leads that can be pursued in the present data set comprising lipid mediator plasma concentrations acquired from a small sample of patients with different psychiatric diagnoses or controls. Following data transformation and application of unsupervised and supervised methods of data exploration and modeling, a hypothesis could be obtained that pointed at patients with dementia as a group of psychiatric patients that displays the most distinct lipid mediator plasma concentration pattern among a mixed cohort of healthy subjects and patients with depression, bipolar disorder or ADHD. Although the present data set was small and with diagnostic accuracies of only close to 80% a useful biomarker was not obtained, results nevertheless encourage further research on a plasma lipid mediator concentration-based complex biomarker for dementia. Whether others of the included psychiatric diseases also display distinct lipid mediator patterns is discouraged by the present results, however, considering the small sample size this cannot be excluded. For dementia, among 35 candidate lipid mediators, a subset of seven lipid mediators provided a better diagnostic performance than similarly sized sets of randomly chosen lipid mediators.

In the present study, a targeted approach to quantify lipid mediators was chosen because it was expected to provide a more robust numerical basis for testing of the possibility to develop a lipid mediator based biomarker in psychiatric diseases than an untargeted approach (2, 6, 7). In addition, it was non-redundant to previous untargeted approaches conducted for investigating lipid profiles in dementia/Alzheimer's disease (10, 11), depression (12), and bipolar disorder (12). In these studies lipids of several different classes were investigated including lysophosphatidylcholine, lysophosphatidylethanolamine, phosphatidylcholine, phosphatidylethanolamine, phosphatidylinositols, sphingomyelins, diacylglycerol, and triacylglycerol but not covering the group of lipid mediators present in lower concentrations. Alterations in the lipid metabolism were found in dementia/Alzheimer's disease, depression and bipolar disorder, supporting lipidomics as a reasonable, and promising approach to biomarker creation for neurological disorders. However, a final biomarker composition was not provided while several lipids were suggested to merit further studies. Among the rarer targeted approaches to the detection of lipid mediators in neuropsychiatric patients is a report of an altered sphingolipid metabolism in Alzheimer's disease (70), which is consistent to the present results that suggest identifying patients with dementia using seven lipid mediators, all belonging to the group of sphingolipids.

## Conclusions

Aim of this study was the creation of a (complex) biomarker for the differentiation of a psychiatric disease within a group of patients with different psychiatric diseases namely depression, bipolar disorder, attention-deficit hyperactivity disorder, or dementia and a control group. No concrete hypothesis like a single metabolic pathway was taken as basis for the selection of analytes but a broad range of 41 lipid mediators and other metabolites was analyzed. Using a data-science based approach the identification of patients diagnosed with dementia in the examined group by a complex biomarker including seven lipid mediators was possible. Because of the limited number of patients further investigations including higher numbers of participants as well as an age-matched control group are necessary.

## Author Contributions

AR and GG conceived and designed the experiments. SE, DP, and AR conducted the clinical part of the study and collected blood. RG, DT, and SF processed the samples and assayed the lipid concentrations. RG performed data and preparation for bioinformatics analysis. JL conceived, programmed, and performed the data analysis. RG, JL, and DP wrote the paper. GG and AR discussed the manuscript. All authors have approved the final version of the manuscript.

### Conflict of Interest Statement

The authors declare that the research was conducted in the absence of any commercial or financial relationships that could be construed as a potential conflict of interest.
