# Supplementary material for: A Data Science-Based Analysis Points at Distinct Patterns of Lipid Mediator Plasma Concentrations in Patients With Dementia
Source: Front Psychiatry. 2019 Feb 11;10:41. doi: 10.3389/fpsyt.2019.00041 (PMC6378270; doi:10.3389/fpsyt.2019.00041)
Supplement: Supplementary file 1 [file Data_Sheet_1.ZIP › Supplementary_Methods_1.docx]

**Ceramides and sphingolipids**

**Reference substances**

Analytes

Sphingosine – Avanti Polar Lipids, Alabaster, AL, USA

Sphingosine-1-phosphate – Avanti Polar Lipids, Alabaster, AL, USA

Sphinganine-1-phosphate – Avanti Polar Lipids, Alabaster, AL, USA

C16 Sphinganine – Avanti Polar Lipids, Alabaster, AL, USA

C18 Sphinganine – Avanti Polar Lipids, Alabaster, AL, USA

C24 Sphinganine – Avanti Polar Lipids, Alabaster, AL, USA

C24:1 Sphinganien – Avanti Polar Lipids, Alabaster, AL, USA

C14 Cer – Avanti Polar Lipids, Alabaster, AL, USA

C16 Cer – Avanti Polar Lipids, Alabaster, AL, USA

C18 Cer – Avanti Polar Lipids, Alabaster, AL, USA

C20 Cer – Avanti Polar Lipids, Alabaster, AL, USA

C24 Cer – Avanti Polar Lipids, Alabaster, AL, USA

C24:1 Cer – Avanti Polar Lipids, Alabaster, AL, USA

C16 GluCer – Avanti Polar Lipids, Alabaster, AL, USA

C18 GluCer – Avanti Polar Lipids, Alabaster, AL, USA

C18:1 GluCer – Avanti Polar Lipids, Alabaster, AL, USA

C24:1 GluCer – Avanti Polar Lipids, Alabaster, AL, USA

C16 LacCer – Avanti Polar Lipids, Alabaster, AL, USA

C18 LacCer – Avanti Polar Lipids, Alabaster, AL, USA

C24 LacCer – Avanti Polar Lipids, Alabaster, AL, USA

C24:1 LacCer – Avanti Polar Lipids, Alabaster, AL, USA

Working solutions for the generation of calibrator and QC-samples were prepared as a mixture of all analytes by serial dilution using a mixture of tetrahydrofuran and chloroform.

Internal Standard

SPH-d_7_ – Avanti Polar Lipids, Alabaster, AL, USA

S1P-d_7_ – Avanti Polar Lipids, Alabaster, AL, USA

C18-Sphinganine-d_3_ – Avanti Polar Lipids, Alabaster, AL, USA

C16Cer-d_7_ – Avanti Polar Lipids, Alabaster, AL, USA

C18Cer-d_3_ – Avanti Polar Lipids, Alabaster, AL, USA

C24Cer-d_4_ – Chiroblock GmbH, Wolfen, Germany

C24:1Cer-d_7_ – Avanti Polar Lipids, Alabaster, AL, USA

C18GluCer-d_5_ – Avanti Polar Lipids, Alabaster, AL, USA

C16LacCer-d_3_ – Avanti Polar Lipids, Alabaster, AL, USA

C17LacCer – Avanti Polar Lipids, Alabaster, AL, USA

Working solution for the internal standard was prepared as a mixture of all internal standards by serial dilution using a mixture of tetrahydrofuran and chloroform.

**Instrumentation**

Mass spectrometer QTrap 5500 (Sciex, Darmstadt, Germany)

Turbo-V-source in positive ESI mode

HPLC Agilent Technologies 1200 series (Agilent, Waldbronn, Germany)

G1312B – 1260 BinPump

G1379B – 1200 Vacuum Degasser

G1316B – 1200 TCC SL

G1310A – 1100 isocratic pump

HTC Pal autosampler (Chromtech, Idstein, Germany)

LC-column Zorbax Eclipse Plus C18 50 mm x 2.1 mm ID, 1.8 μm (Agilent technologies, Waldbronn, Germany)

Precolumn: Luna C18, Phenomenex, Aschaffenburg, Germany

Solvent A 2 mM ammonium formate in water + 0.2% formic acid

Solvent B acetonitrile/isopropanol/acetone (50/30/20, v/v/v) + 0.2% formic acid

Solvent C acetonitrile + 0.1% formic acid (was infused post column by an isocratic pump)

Injection volume 10 µL

Gradient

Time flow rate (µL/min) percent solvent A percent solvent B

0,00 400 60 40

0,60 400 60 40

4,20 400 0 100

6,00 400 0 100

6,50 300 0 100

9,00 300 0 100

15,00 400 0 100

16,00 400 60 40

23,00 400 60 40

**Sample preparation and extraction**

Plasma samples were thawed at room temperature and processed as follows using liquid-liquid-extraction:

1. 10 µL sample
2. Add 20 µL internal standard solution
3. Add 100 µL water
4. Add 100 µL extraction buffer (citric acid 30 mM, disodium hydrogen phosphate 40 mM)
5. Extract mixture once with 600 µl methanol/chloroform/hydrochloric acid (15:83:2, v/v/v)
6. Remove lower organic phase and evaporate it at 45°C under a gentle stream of nitrogen
7. Reconstituted in 200 µl of tetrahydrofuran/water (9:1, v/v) with 0.2% formic acid and 10 mM ammonium formate.

For the preparation of calibration standards and quality control samples, 20 µL of a working solution were processed as stated instead of 10 µL sample.

The analysis was done in Multiple Reaction Monitoring (MRM) mode. Information on the used precursor to product ion transitions (m/z), used internal standards as well as the lower and upper limit of quantification can be found in Table 1. Data Acquisition was done using Analyst Software V 1.6.2 and quantification was performed with MultiQuant Software 3.0.2 (both Sciex, Darmstadt, Germany), employing the internal standard method (isotope dilution mass spectrometry). Calibration curves were calculated by linear or quadratic regression with 1/x or 1/x^2^ weighting.

Table 1: Ceramides and sphingolipids

| Analyt | Q1 | Q3 (Quan/Qual) | IS | LLOQ (ng/mL) | ULOQ (ng/mL) |
| --- | --- | --- | --- | --- | --- |
| Sphingosine | 300,3 | 282.3 / 252.3 | SPH-d_7_ | 0.6 | 300 |
| Sphingosine-1-phosphate | 380,2 | 264.2 / 362.3 | S1P-d_7_ | 4 | 2000 |
| Sphinganine-1-phosphate | 382,3 | 284.1 / 266.3 | S1P-d_7_ | 1 | 500 |
| C16 Sphinganine | 540.5 | 522.5 / 284.3 | C18-Sphinganine-d_3_ | 1 | 500 |
| C18 Sphinganine | 568.6 | 550.6 / 284.3 | C18-Sphinganine-d_3_ | 2 | 500 |
| C24 Sphinganine | 652.7 | 284.2 / 634.2 | C18-Sphinganine-d_3_ | 4 | 2000 |
| C24:1 Sphinganine | 650.6 | 284.2 / 632.6 | C18-Sphinganine-d_3_ | 4 | 2000 |
| C14 Cer | 510.5 | 264.2 / 492.5 | C16Cer-d_7_ | 1 | 500 |
| C16 Cer* | 539.5 | 264.2 / 521.4 | C16Cer-d_7_ | 32 | 16000 |
| C18 Cer* | 567.5 | 264.4 / 549.5 | C18Cer-d_3_ | 4 | 2000 |
| C20 Cer | 594.6 | 264.4 / 576.5 | C18Cer-d_3_ | 4 | 2000 |
| C24 Cer* | 651.6 | 264.2 / 633.6 | C24Cer-d_4_ | 32 | 16000 |
| C24:1 Cer* | 649.6 | 264.2 / 631.6 | C24:1Cer-d_7_ | 32 | 16000 |
| C16 GluCer | 701.6 | 264.2 / 683.5 | C18GluCer-d_5_ | 32 | 16000 |
| C18 GluCer | 728.6 | 710.6 / 264.2 | C18GluCer-d_5_ | 1 | 500 |
| C18:1 GluCer | 726.6 | 264.2 / 708.5 | C18GluCer-d_5_ | 1 | 500 |
| C24:1 GluCer* | 811.7 | 264.3 / 793.7 | C24Cer-d_4_ | 32 | 16000 |
| C16 LacCer* | 863.6 | 264.3 / 521.5 | C16LacCer-d_3_ | 32 | 9600 |
| C18 LacCer | 890.6 | 264.2 / 548.5 | C17LacCer | 1 | 500 |
| C24 LacCer | 974.7 | 264.3 / 632.6 | C24Cer-d_4_ | 32 | 16000 |
| C24:1 LacCer | 973.7 | 264.2 / 631.5 | C24Cer-d_4_ | 32 | 16000 |

*because of high signal intensity the ^13^C-isotope was measured.

**Lysophosphatidic acids**

**Reference substances**

Analytes

LPA 16:0 – Avanti Polar Lipids, Alabaster, AL, USA

LPA 18:0 – Avanti Polar Lipids, Alabaster, AL, USA

LPA 18:1 – Echelon Biosciences Inc, Salt Lake City, UT, USA

LPA 18:2 – Echelon Biosciences Inc, Salt Lake City, UT, USA

LPA 20:4 – Echelon Biosciences Inc, Salt Lake City, UT, USA

Working solutions for the generation of calibrator and QC-samples were prepared as a mixture of all analytes by serial dilution using methanol.

Internal Standard

LPA 17:0 – Avanti Polar Lipids, Alabaster, AL, USA

Working solution for the internal standard was prepared by serial dilution using methanol.

**Instrumentation**

Mass spectrometer QTrap 5500 (Sciex, Darmstadt, Germany)

Turbo-V-source in negative ESI mode

HPLC Agilent Technologies 1200 series (Agilent, Waldbronn, Germany)

G1312B – 1260 BinPump

G1379B – 1200 Vacuum Degasser

G1316B – 1200 TCC SL

HTC Pal autosampler (Chromtech, Idstein, Germany)

LC-column C18 Mercury 20 x 2 mm, 3 µm, 100 Å (Phenomenex, Aschaffenburg, Germany)

Precolumn: Luna C18 (Phenomenex, Aschaffenburg, Germany)

Solvent A 50 mM ammonium formate in water + 0.2% formic acid

Solvent B acetonitrile/isopropanol (50/50, v/v) + 0.2% formic acid

Injection volume 20 µL

Gradient

Time flow rate (µL/min) percent solvent A percent solvent B

0,00 400 60 40

0,50 400 60 40

1,00 400 5 95

3,50 400 5 95

4,00 400 60 40

7,00 400 60 40

**Sample preparation and extraction**

Plasma samples were thawed at room temperature and processed as follows using liquid-liquid-extraction:

1. 50 µL sample
2. Add 10 µL methanol
3. Add 20 µL internal standard solution
4. Add 800 µL methanol/0.1 M HCl in water (50:50, v/v)
5. Vortex
6. Extract sample twice using 400 µl chloroform
7. Combine organic phases and evaporate the organic phase at 45 °C under a gentle stream of nitrogen
8. Reconstitute sample in 200 µL methanol

Phosphate-buffered-saline (PBS) was used for the preparation of calibration standards and quality control samples. A volume of 50 µl PBS was combined with 10 µL of a working solution and processed as stated before starting at step 3.

The analysis was done in Multiple Reaction Monitoring (MRM) mode. Information on the used precursor to product ion transitions (m/z), used internal standards as well as the lower and upper limit of quantification can be found in Table 2. Data Acquisition was done using Analyst Software V 1.6.2 and quantification was performed with MultiQuant Software 3.0.2 (both Sciex, Darmstadt, Germany), employing the internal standard method (isotope dilution mass spectrometry). Calibration curves were calculated by linear regression with 1/x weighting.

Table 2: Lysophosphatidic acids

| Analyt | Q1 | Q3 (Quan/Qual) | IS | LLOQ (ng/mL) | ULOQ (ng/mL) |
| --- | --- | --- | --- | --- | --- |
| LPA 16:0 | 409.2 | 153.0 / 255.2 | LPA 17:0 | 1 | 500 |
| LPA 18:0 | 437.2 | 153.0 / 283.3 | LPA 17:0 | 1 | 500 |
| LPA 18:1 | 435.2 | 153.0 / 281.2 | LPA 17:0 | 2,5 | 500 |
| LPA 18:2 | 433.2 | 153.0 / 79.0 | LPA 17:0 | 1 | 500 |
| LPA 20:4 | 457.2 | 153.0 / 79.0 | LPA 17:0 | 2,5 | 500 |

**Cyclic nucleotides**

**Reference substances**

3‘,5‘-cGMP Sigma Aldrich, Steinheim, Germany

3‘,5‘-cAMP Sigma Aldrich, Steinheim, Germany

Working solutions for the generation of calibrator and QC-samples were prepared as a mixture of all analytes by serial dilution using methanol.

^13^C_5_-cAMP Toronto Research Chemical, North York, ON, Canada

Working solution for the internal standard was prepared by serial dilution using methanol.

**Instrumentation**

Mass spectrometer QTRAP 5500 (Sciex, Darmstadt, Germany)

Turbo-V-source in negative ESI mode

HPLC Agilent Technologies 1200 series (Agilent, Waldbronn, Germany)

G1312B – 1260 BinPump

G1379B – 1200 Vacuum Degasser

G1316B – 1200 TCC SL

HTC Pal autosampler (Chromtech, Idstein, Germany)

LC-column Atlantis T3 3µm 100x2,1mm (Waters, Eschborn, Germany)

AQ C18 guard column 4 mm, 2 mm I.D. (Phenomenex, Aschaffenburg, Germany)

Solvent A Water + 0,1% acetic acid

Solvent B 10 mM ammonium formate in methanol

Injection volume 15 µL

Gradient

Time flow rate (µL/min) percent solvent A percent solvent B

0,00 300 100 0

1,00 300 100 0

4,50 300 15 85

8,00 300 15 85

8,10 300 100 0

12,00 300 100 0

**Sample preparation and extraction**

Plasma samples were thawed at room temperature and processed as follows using liquid-liquid-extraction:

1. 50 µL sample
2. Add 20 µL methanol
3. Add 40 µL IS solution
4. Add 340 µL methanol
5. Vortex and centrifuge sample (20,238g for 3.5 min)
6. Remove supernatant and evaporate it at 45 °C under a gentle stream of nitrogen
7. Reconstitute sample in 50 µL water + 0,1% acetic acid

Artificial plasma was used for the preparation of calibration standards and quality control samples. Artifical plasma was prepared as described in Kij et al., (2016) without adding albumin. A volume of 50 µl artificial plasma was combined with 20 µL of a working solution and processed as stated before starting at step 3.

The analysis was done in Multiple Reaction Monitoring (MRM) mode. Information on the used precursor to product ion transitions (m/z), used internal standards as well as the lower and upper limit of quantification can be found in Table 3. Data Acquisition was done using Analyst Software V 1.6.2 and quantification was performed with MultiQuant Software V 3.0.2 (both Sciex, Darmstadt, Germany), employing the internal standard method (isotope dilution mass spectrometry. Calibration curves were calculated by linear regression with 1/x^2^ weighting.

Table 3: Cyclic nucleotides

| Analyt | Q1 | Q3 (Quan/Qual) | IS | LLOQ (ng/mL) | ULOQ (ng/mL) |
| --- | --- | --- | --- | --- | --- |
| 3’,5’-cGMP | 344,0 | 150,0 / 133,0 | ^13^C_5_-cAMP | 0,48 | 200 |
| 3’,5’-cAMP | 328,0 | 134,0 / 79,0 | ^13^C_5_-cAMP | 0,48 | 200 |

Kij, A. et al. “Simultaneous quantification of PGI 2 and TXA 2 metabolites in plasma and urine in NO-deficient mice by a novel UHPLC/MS/MS method.” J. Pharm. Biomed. Anal. 129, 148–154 (2016).

**Nucleosides and** **nucleoside triphosphates**

**Reference substances**

GTP Sigma Aldrich, Steinheim, Germany

ATP Sigma Aldrich, Steinheim, Germany

Working solutions for the generation of calibrator and QC-samples were prepared as a mixture of all analytes by serial dilution using methanol.

Cytidine Sigma Aldrich, Steinheim, Germany

2deoxycytidine Sigma Aldrich, Steinheim, Germany

Guanosine Sigma Aldrich, Steinheim, Germany

Thymidine Sigma Aldrich, Steinheim, Germany

Uridine   Sigma Aldrich, Steinheim, Germany

Working solutions for the generation of calibrator and QC-samples were prepared as a mixture of all analytes by serial dilution using methanol.

^13^C_10_, ^15^N_5_-GTP Sigma Aldrich, Steinheim, Germany

^13^C_10_, ^15^N_5_-ATP Sigma Aldrich, Steinheim, Germany

^13^C_5_-Cytidine Omicron Biochemicals, South Bend, USA

^13^C, ^15^N_3_-2deoxycytidine Alsachim, Illkirch Graffenstaden, France

^13^C_5_-Guanosine Alsachim, Illkirch Graffenstaden, France

^13^C_5_-Adenosine Alsachim, Illkirch Graffenstaden, France

^13^C_5_- Uridine  Alsachim, Illkirch Graffenstaden, France

Working solution for the internal standard was prepared by serial dilution using methanol.

**Instrumentation – measurement of nucleoside triphosphates**

Mass spectrometer QTRAP 5500 (Sciex, Darmstadt, Germany)

Turbo-V-source in positive ESI

HPLC Agilent Technologies 1200 series (Agilent, Waldbronn, Germany)

G1312B – 1260 BinPump

G1379B – 1200 Vacuum Degasser

G1316B – 1200 TCC SL

HTC Pal autosampler (Chromtech, Idstein, Germany)

LC-column BioBasic AX column 150 mm×2.1mm, 5 μm particle size (Thermo Fisher, Waltham, MA, USA)

Precolumn: BioBasic AX (Thermo Fisher, Waltham, MA, USA)

Solvent A 400 mL acetonitrile + 0.6 mL glacial acetic acid + 10 mL ammonium acetate in water (1 M) + 590 mL water

Solvent B 315 mL acetonitrile + 3,15 mL ammonia solution (25 %) + 1,05 mL ammonium acetate in water (1 M) + 708 mL water

Injection volume 20 µL

Gradient

Time flow rate (µL/min) percent solvent A percent solvent B

0,00 450 70 30

4,00 450 30 70

11,00 450 10 90

12,00 450 10 90

12,50 450 70 30

15,00 450 70 30

**Instrumentation – measurement of nucleosides**

Mass spectrometer Qtrap 5500 (Sciex, Darmstadt, Germany)

Turbo-V-source in positive ESI mode

HPLC Agilent Technologies 1200 series (Agilent, Waldbronn, Germany)

G1312B – 1260 BinPump

G1379B – 1200 Vacuum Degasser

G1316B – 1200 TCC SL

G1310A – 1100 isocratic pump

HTC Pal autosampler (Chromtech, Idstein, Germany)

LC-column Atlantis T3 3µm 100x2,1mm (Waters, Eschborn, Germany)

AQ C18 guard column 4 mm, 2 mm I.D. (Phenomenex, Aschaffenburg, Germany)

Solvent A Water + 0,1% acetic acid

Solvent B 10 mM ammonium acetate in methanol

Solvent C Isopropanol (was infused post column by an isocratic pump)

Injection volume 20 µL

Gradient

Time flow rate (µL/min) percent solvent A percent solvent B

0,00 300 100 0

2,50 300 100 0

5,00 300 10 90

9,50 300 10 90

11,00 300 100 0

15,00 300 100 0

**Sample preparation and extraction**

A combined internal standard solution containing IS for nucleosides and nucleoside triphosphates was used in the sample preparation. Samples were split after the extraction and measured with separate methods for nucleosides and nucleoside triphosphates, respectively. Standards and QC-samples were prepared separately for the two groups of analytes.

Plasma samples were thawed at room temperature and processed as follows using protein precipitation:

1. 50 µL sample
2. Add 20 µL methanol
3. Add 20 µL IS solution
4. Add 340 µL methanol
5. Vortex and centrifuge
6. Remove supernatant and and split supernatant (180 µL for determination of Nuk and NTP, respectively)
7. Evaporate the organic phase at 45 °C under a gentle stream of nitrogen
8. Reconstitute sample in:
   1. Nuk: 50 µL water + 0,1% acetic acid
   2. NTP: 50 µL mixture of solvent A and B (70:30, v/v)

Artificial plasma was used for the preparation of calibration standards and quality control samples. Artifical plasma was prepared as described in Kij et al., (2016) without adding albumin. A volume of 50 µl artificial plasma was combined with 20 µL of a working solution and processed as stated before starting at step 3. Standard and QC-samples were reconstituted in 100 µL reconstitution solvent instead of 50 µL.

The analysis was done in Multiple Reaction Monitoring (MRM) mode. Information on the used precursor to product ion transitions (m/z), used internal standards as well as the lower and upper limit of quantification can be found in Table 4 and Table 5. Data Acquisition was done using Analyst Software 1.6.2 and quantification was performed with MultiQuant Software 3.0.2 (both Sciex, Darmstadt, Germany), employing the internal standard method (isotope dilution mass spectrometry). Calibration curves were calculated by linear regression with 1/x weighting.

Table 4: Nucleoside triphosphates

| Analyt | Q1 | Q3 (Quan/Qual) | IS | LLOQ (ng/mL) | ULOQ (ng/mL) |
| --- | --- | --- | --- | --- | --- |
| GTP | 524,0 | 152,0 / 135,0 | ^13^C_10_, ^15^N_5_-GTP | 20 | 5000 |
| ATP | 508,0 | 410,0 / 136,1 | ^13^C_10_, ^15^N_5_-ATP | 50 | 25000 |

Table 5: Nucleosides

| Analyt | Q1 | Q3 (Quan/Qual) | IS | LLOQ (ng/mL) | ULOQ (ng/mL) |
| --- | --- | --- | --- | --- | --- |
| Cytidine | 244,2 | 112,1 / 94,9 | ^13^C_5_-Cytidine | 0,5 | 250 |
| dCytidine | 228,1 | 112,0 / 95,0 | ^13^C, ^15^N_3_-  2deoxycytidine | 0,2 | 100 |
| Guanosine | 284.0 | 152.0 / 135.1 | ^13^C_5_-Guanosine | 0,2 | 100 |
| Thymidine | 243,1 | 127,0 / 110,0 | ^13^C_5_-Adenosine | 0,5 | 250 |
| Uridine | 245,0 | 113,0 / 96,0 | ^13^C_5_- Uridine | 4 | 2000 |

Kij, A. et al. “Simultaneous quantification of PGI 2 and TXA 2 metabolites in plasma and urine in NO-deficient mice by a novel UHPLC/MS/MS method.” J. Pharm. Biomed. Anal. 129, 148–154 (2016).

**Endocannabinoids and structurally related compounds**

**Reference substances**

Arachidonoyl Ethanolamide (AEA) Cayman Chemical Company, Ann Arbor, MI, USA

Palmitoyl Ethanolamide (PEA) Cayman Chemical Company, Ann Arbor, MI, USA

Oleoyl Ethanolamide (OEA) Cayman Chemical Company, Ann Arbor, MI, USA

Docosahexaenoyl Ethanolamide (DHEA) Cayman Chemical Company, Ann Arbor, MI, USA

1-arachidonoyl glycerol (1-AG) Cayman Chemical Company, Ann Arbor, MI, USA

2-arachidonoyl glycerol (2-AG) Cayman Chemical Company, Ann Arbor, MI, USA

Working solutions for the generation of calibrator and QC-samples were prepared as a mixture of all analytes by serial dilution using acetonitrile.

AEA-d_8_ Cayman Chemical Company, Ann Arbor, MI, USA

PEA-d_4_ Cayman Chemical Company, Ann Arbor, MI, USA

OEA-d_4_ Cayman Chemical Company, Ann Arbor, MI, USA

DHEA-d_4_ Cayman Chemical Company, Ann Arbor, MI, USA

1-AG-d_5_ Cayman Chemical Company, Ann Arbor, MI, USA

2-AG-d_5_ Cayman Chemical Company, Ann Arbor, MI, USA

Working solution for the internal standard was prepared by serial dilution using acetonitrile.

**Instrumentation**

Mass spectrometer QTRAP 6500+ (Sciex, Darmstadt, Germany)

Turbo Ion Spray in positive ESI

HPLC Agilent Technologies 1290 Infinity (Agilent, Waldbronn, Germany)

G4220A – 1290 BinPump

G4226A – 1290 Sampler

G1330B – 1290 Thermostat

G1316C – 1290 TCC

LC-column Zorbax Eclipse Plus C18 50 mm x 2.1 mm ID, 1.8 μm (Agilent technologies, Waldbronn, Germany)

Precolumn: Luna C18 (Phenomenex, Aschaffenburg, Germany)

Solvent A Water + 0,0025% formic acid

Solvent B Acetonitrile + 0,0025% formic acid

Injection volume 10 µL

Gradient

Time flow rate (µL/min) percent solvent A percent solvent B

0,00 350 75 25

0,50 350 75 25

1,00 350 25 75

2,25 350 12 88

4,40 350 12 88

4,50 350 75 25

6,00 350 75 25

**Sample preparation and extraction**

Plasma samples were thawed at room temperature and processed as follows using liquid-liquid-extraction:

1. 50 µL sample
2. Add 50 µL acetonitrile
3. Add 50 µL IS solution
4. Vortex
5. Extract sample twice using 50 µL and 150 µL ethylacetate/hexane (90:10 – v:v), respectively
6. Combine organic phases and evaporate the organic phase at 45 °C under a gentle stream of nitrogen
7. Reconstitute sample in 50 µL acetonitrile

Phosphate-buffered-saline (PBS) was used for the preparation of calibration standards and quality control samples. A volume of 50 µl PBS was combined with 50 µL of a working solution and processed as stated before starting at step 3.

The analysis was done in Multiple Reaction Monitoring (MRM) mode. Information on the used precursor to product ion transitions (m/z), used internal standards as well as the lower and upper limit of quantification can be found in Table 6. Data Acquisition was done using Analyst Software 1.6.3 and quantification was performed with MultiQuant Software 3.0.2 (both Sciex, Darmstadt, Germany), employing the internal standard method (isotope dilution mass spectrometry). Calibration curves were calculated by linear regression with 1/x weighting.

Table 6: Endocannabinoids and structurally related compounds

| Analyt | Q1 | Q3 (Quan/Qual) | IS | LLOQ (ng/mL) | ULOQ (ng/mL) |
| --- | --- | --- | --- | --- | --- |
| AEA | 348,3 | 287,3 / 91,0 | AEA-d_8_ | 0,05 | 10 |
| PEA | 300,2 | 62,0 / 95,0 | PEA-d_5_ | 0,25 | 50 |
| OEA | 326,3 | 62,0 / 309,2 | OEA-d_4_ | 0,25 | 50 |
| DHEA | 372.2 | 311.2 / 131.0 | DHEA-d_4_ | 0,05 | 10 |
| 1-AG | 379.2 | 287.3 / 269.2 | 1-AG-d_5_ | 0,50 | 100 |
| 2-AG | 379.2 | 287.3 / 269.2 | 2-AG-d_5_ | 0,50 | 100 |
